# Serious Inadequacies in High Alert Medication-Related Knowledge Among Pakistani Nurses: Findings of a Large, Multicenter, Cross-sectional Survey

**DOI:** 10.3389/fphar.2020.01026

**Published:** 2020-07-14

**Authors:** Muhammad Salman, Zia Ul Mustafa, Alina Zeeshan Rao, Qurat-ul-Ain Khan, Noman Asif, Khalid Hussain, Naureen Shehzadi, Muhammad Farhan Ali Khan, Amir Rashid

**Affiliations:** ^1^ Department of Pharmacy Practice, Faculty of Pharmacy, The University of Lahore, Lahore, Pakistan; ^2^ Department of Pharmacy, District Headquarter Hospital, Pakpattan, Pakistan; ^3^ Punjab University College of Pharmacy, University of the Punjab, Lahore, Pakistan; ^4^ Department of Pharmacy, Punjab Institute of Cardiology, Lahore, Pakistan; ^5^ Department of Pharmacy, Quaid-i-Azam University, Islamabad, Pakistan; ^6^ Department of Pharmacology, Faculty of Pharmacy, The University of Lahore, Islamabad, Pakistan

**Keywords:** barriers, high alert medications, knowledge, nurses, Pakistan

## Abstract

**Introduction:**

Deaths-related to medications errors are common in Pakistan but these are not accurately reported. Recently, the death of a 9 months old baby due to abrupt administration of 15% potassium chloride injection sparked the issue of high alert medications (HAMs) related errors in the country. Since drug administration is the prime responsibility of the nurses, it is pivotal that they possess good knowledge of HAMs. Since there is no published data regarding the knowledge of HAMs among Pakistani nurses, we aimed to assess knowledge of HAMs among registered nurses of Pakistan.

**Methods:**

A cross-sectional study was conducted among registered nurses, recruited using a convenient sampling technique, from 29 hospitals all over the Punjab Province. Data were collected using a validated self-administered instrument. All data were entered and analyzed using SPSS version 22.

**Results:**

The study sample was comprised of 2,363 registered nurses (staff nurses = 94.8%, head nurses = 5.2%). Around 63% were working in tertiary hospitals whereas almost 25 and 12% were from district headquarter hospitals and tehsil headquarter hospitals, respectively. Around 84% of the study participants achieved scores <70%, indicating majority of Pakistani nurses having poor knowledge of HAMs administration as well as regulation. There was no significant difference of overall knowledge among age, hospitals, departments, training, designations, qualification, and experience categories. Major obstacles encountered during HAMs administration were “getting uncertain answers from colleagues” (72.9%), “unavailability of suitable person to consult” (61.1%) and “receiving verbal orders” (55.6%).

**Conclusion:**

Our study revealed the serious inadequacies in HAMs knowledge among Pakistani nurses which may lead to adverse patient outcomes. Nurses should receive comprehensive pharmacology knowledge not only during in-school nursing education but also as hospital-based continuing education. Moreover, it is of immense importance to bridge the gaps between physicians, clinical pharmacists, and nurses through effective communication as this will help reduce medication errors and improve patient care.

## Introduction

Medication error is defined as “any preventable event that may cause or lead to inappropriate medication use or patient harm while the medication is in the control of the health professional, patient, or consumer. Such events may be related to professional practice, health care products, procedures, and systems, including prescribing, order communication, product labeling, packaging, and nomenclature, compounding, dispensing, distribution, administration, education, monitoring, and use” (NCCMERP, 2015). There are several ways to classify these errors. One way is to classify these errors in the sequence of medication use process: prescribing, transcribing, dispensing, administration, or monitoring (WHO, 2016). Another approach is to classify them according to whether they occur from mistakes in planning actions (knowledge-based or rule-based errors) or errors in carrying out an action (action-based errors, also called “slips,” or memory-based errors, also called “lapses”) (Ferner and Aronson, 2006; Aronson, 2009). A further approach is to classify medication errors as wrong patient, medication, dose, frequency, duration, and route of administration (WHO, 2016). Medication errors pose life threatening risk to the patient and a high economic loss (Perrone et al., 2014; Tshiamo et al., 2015; Akhideno et al., 2018; Formica et al., 2018). Medication error is the prime patient safety and quality care concern nowadays. Globally, 2–5% hospital admissions are due to medication errors and most of them are preventable (Roughead et al., 2013; Perrone et al., 2014; AHRQ, 2015; Latimer et al., 2017; Iftikhar et al., 2018). According to the statistics of Centers for Disease Control and Prevention, medications errors are the third leading cause of mortality in United States with 98,000 deaths per annum (Pham et al., 2012; Makary and Daniel, 2016).

High alert medications (HAMs) cause serious health injuries or even death to the patient if use improperly. As compared to ordinary drugs, HAMs carry greater risks of adverse events (Labib et al., 2018; Shen et al., 2018). The American Pharmaceutical Association categorizes HAMs into following classes, chemotherapeutic agents, cardiovascular medications, anticoagulants, opioids and their derivatives, neuromuscular blocking agents, benzodiazepines, and some electrolytes like potassium chloride (15%) (Zyoud et al., 2019). Majorly, these medications are used in acute and emergency rooms. Others places of HAMs usage are intensive care unit, coronary care unit, hemodialysis unit, pediatrics ward, medical ward, as well in labor room and surgical ward.

Nurses are the nucleus of health care system. Being a vital member of health care provision team, they are actively involved in patient care. Administration of drug to the particular patient according to the prescriber’s order is one of the most crucial tasks performed by nursing staff. Unlike the other health care team members, nurses are always in close contact with the patient throughout the course of their treatment. Therefore, they are well aware about the actual condition of patient before and after drug administration and can actively report adverse drug reactions. The most common nursing-associated medication errors are related to administrations of drugs (Choi et al., 2016). Published data showed that the major reasons of medication administration errors by nurses are related to medication packaging e.g. similarities in packaging, appearance and names (Mrayyan et al., 2007; Dumo, 2012; Mrayyan, 2012; Hammoudi et al., 2018), and poor communication between physicians and nurses (Kim et al., 2011; Dumo, 2012; Topcu et al., 2017; Hammoudi et al., 2017). With regards to the barriers of medication errors reporting, important factors are the administrator’s responses to medication errors, fear, reporting efforts, disagreements on the definitions of errors (Chiang et al., 2010; Petrova et al., 2010; Kim et al., 2011; Aboshaiqah, 2013; Shamim et al., 2016; Hammoudi et al., 2018). Pakistan is a resource-limited country where disease burden is very high. The current status of country’s health care system is worrying. Deaths due to medications errors are common but these are not reported due to the lack of required mechanism and fear of patient relatives’ reaction or adverse career-related repercussions (DAWN News, 2017). Recently, the death of a 9 months old baby in a private sector hospital of Karachi (Capital city of Sindh, Pakistan) due to abrupt administration of 15% potassium chloride (KCl) injection had highlighted the issue of high alert related medication errors in the country (Jafferi, 2019; Geo News, 2019; 92 News, 2019). According to the Pakistan economic survey 2017–2018, 108,474 registered nurses were performing duties across the country (FDGOP, 2019). Their awareness about the high alert medication is of vital importance as these drugs possess greater potential to cause serious consequences, if administered inappropriately. To the best of our knowledge and literature survey, there is no published data regarding the knowledge of HAMs among registered Pakistani nurses. Therefore, current study was carried out to estimate the knowledge about administration and regulation of HAMs among registered nurses of Pakistan. Moreover, the possible barriers faced in the administration of HAMs were also evaluated.

## Methods

### Study Design and Setting

This cross-sectional study was conducted among the registered nurses from four metropolitan divisions of province Punjab namely Sahiwal, Multan, Faisalabad, and Lahore. The data were obtained from 29 health care settings (Secondary and tertiary care hospitals), including 11 tertiary/teaching hospitals, 9 District Head Quarter (DHQ) hospitals, and 9 Tehsil Head Quarter (THQ) hospitals during a period of 6 months (Feb–July 2019). A convenient sampling technique was employed and trained investigators approached the nursing staff in the above-mentioned hospitals and briefed them about the intent of this study. Those who were willing to be enrolled were administered the study questionnaire.

### Ethical Approval

Protocol of the current study was reviewed and approved by the Research Ethics Committee of the Department of Pharmacy Practice, Faculty of Pharmacy, The University of Lahore (REC/DPP/FOP/10A). Additionally, written requests, along with documents, were submitted and approvals were obtained from relevant authorities at study sites. The research was conducted in accordance with ethical principles laid down in the 1964 Declaration of Helsinki and its later amendments. As the committee waived the need for written consent, a verbal informed consent was obtained from every nurse before the enrollment and all the participants were ensured about the confidentiality of their responses.

### Inclusion and Exclusion Criteria

The nursing staff registered under the Pakistan nursing council, currently providing services in the emergency room, intensive care unit, dialysis unit, cardiac ward, pediatrics ward, medical ward, obstetrics and gynecology ward, surgical ward, operation theatre, and labor room of the afore-mentioned public sector hospitals were included in the current study. These wards were selected as they carry high risk of errors. Nursing students or nurses working in private sector as well as in the departments other than those mentioned above were excluded from this study.

### Study Instrument

In the present study, a self-administered questionnaire developed by Hsaio et al. (2010) was used to determine HAM-related knowledge of nurses (**Appendix**). This instrument was in English as all the nursing curriculum in Pakistan are taught in English so there was no need for translation into Urdu language. The study instrument had four sections. Sections 1 had 10-items to gather demographics details. Sections 2 and 3 had 10-items each to assess knowledge of HAM administration and regulation, respectively. Each correct response was given 1 score while incorrect or don’t know response were scored 0. Therefore, total knowledge score of HAM administration and regulation was 10 each. To calculate overall HAM-related knowledge score, each correct answer in sections 2 and 3 (total of 20 questions) was given five points, with a total score of 100; incorrect or don’t know answers were scored zero. Knowledge scores were classified as good knowledge ≥70%, or poor knowledge <70%.

A pilot study was conducted in twenty nurses to check the clarity and understandability of all the questions in the study instrument. All participants responded that questions were clear and they were able to understand them all.

### Statistical Analysis

Categorical data were presented as number with percentages. Results of Shapiro-Wilk test revealed the non-normal distribution of continuous variables. Therefore, continuous variables were presented as medians and 25th and 75th percentiles. Continuous data were compared between two groups using the Mann-Whitney U test, and between more than two groups using the Kruskal-Wallis H test. Categorical data were compared using the chi-square test. All statistical analyses were performed using SPSS version 22 for Windows. A p value of less than 0.05 was considered statistically significant.

## Results

A total of 2,500 registered nurses were approached by the investigators and received completely filled study instrument from 2,363 with a response rate of 94.5%.

### Demographic Characteristics

Demographic data of the study participants are shown in **Table 1**. Our study sample was comprised of only females (staff nurses = 94.8%, head nurses = 5.2%), with majority of nurses between 26 and 30 years (36.1%) of age followed by 31–35 years (29.2%). Around 63% were working in teaching hospitals whereas almost 25 and 12% from DHQ and THQ hospitals, respectively. Majority of nurses (37%) were providing services in the Acute & Emergency departments followed by cardiology (16.4%) and gynecology (11.4%).

**Table 1:** Demographic details of the study participants.

Variables	N	%
**Age (years)**		
≤25	494	20.9
>25–30	852	36.1
>30–35	689	29.2
>35	328	13.9
**Hospital type**		
THQ hospital	292	12.4
DHQ hospital	588	24.9
Teaching hospital	1,483	62.8
**Working department**		
Cardiac	387	16.4
Dialysis	148	6.3
Emergency	875	37.0
Obstetrics and gynecology	277	11.7
Labor room	87	3.7
Medical	238	10.1
Operation theater	195	8.2
Pediatric	140	5.9
Surgery	16	0.7
**Training**		
Basic life support	164	6.9
Emergency room training	414	17.5
Infection control	477	20.2
Midwifery	167	7.1
Operation theater	77	3.3
Personal protective equipment	169	7.1
Nursery (Neonatal ICU)	83	3.5
No training	812	34.3
**Qualification**		
Bachelor	2,276	96.3
Master	87	3.7
**Designation**		
Staff nurse	2,240	94.8
Head nurse	123	5.2
**Experience (years)**		
≤2	465	19.7
>2–4	755	32.0
>4–6	228	9.6
>6–8	206	8.7
>8–10	152	6.4
>10	557	23.6

DHQ, District Headquarter; THQ, Tertiary Headquarter.

### Nurses’ Knowledge of High Alert Medication Administration

Responses to the questions related to HAMs administration are presented in **Table 2**. The median (25th and 75th percentile) knowledge score of HAMs administration was 6 (5 and 7). There was no significant difference (P > 0.05) of knowledge scores related to HAMs administration among categories of age, hospitals, departments, training, designations, qualification, and experience.

**Table 2:** Knowledge of High Alert Medications administration.

No.	Questions	Answers	Correct	Incorrect
			N (%)	N (%)
1	Fast intravenous push of 1:1000 epinephrine 1 ampule for patient who has mild allergic reaction	False	1,578 (66.8)	728 (33.2)
2	When an emergency happens, administer 10% calcium chloride (CaCl_2_) 10 ml as a fast intravenous push (in 1–2 minutes)	False	1,726 (66.6)	638 (27.0)
3	10% calcium gluconate and 10% CaCl_2_ are the same drug and are interchangeable	False	1,491 (63.1)	873 (36.9)
4	Dosage expression for insulin injection is “cc” or “ml”	False	1,381 (58.4)	983 (41.6)
5	Accurate chemotherapy dose calculation for adults is based on body weight whereas chemotherapy for children is based on body surface area	False	1,446 (61.2)	917 (38.8)
6	When an emergency such as ventricular fibrillation happens, push fast 15% potassium chloride (KCl) 10 ml intravenously	False	1,502 (63.5)	862 (36.5)
7	15% KCl is better be added to Ringer’s solution for rapid infusion	False	1,485 (62.8)	878 (37.2)
8	Insulin syringe can be replaced by 1 ml syringe	False	942 (39.8)	1,421 (60.1)
9	Give fast IV infusion of 3% NaCl 500 ml for patient who has low sodium level	False	1,298 (54.9)	1,065 (45.1)
10	Port-A route can be used for blood withdrawal and drug injection generally	False	1,431 (60.6)	932 (39.4)

### Nurses’ Knowledge of High Alert Medication Regulation

Participants’ responses to the items regarding the regulation of high risk medications are presented in **Table 3**. The median (25th and 75th percentile) knowledge score of HAMs regulation was 5 (4 and 6). No significant difference of knowledge scores related to HAM regulation was seen among age, departments, training, designations, qualification, and experience categories. However, a statistically significant difference was found among hospital categories. In *post hoc* analysis using Bonferroni correction, only the nurses from DHQ hospitals were found to have significantly less knowledge of HAMs regulation than those working in teaching hospitals (p = 0.003).

**Table 3:** Nurses’ knowledge of high alert medications regulation.

No.	Questions	Answers	Correct	Incorrect
			N (%)	N (%)
1	It is better to use “Amp” or “Vial” for dose expression instead of “mg” or “gm”	False	1,645 (69.6)	718 (30.4)
2	Distinctive labeling should be used on look-alike drugs	True	1,564 (66.2)	799 (33.8)
3	It is right to use “U” instead of unit for dose expression	False	1,834 (77.6)	529 (22.4)
4	For convenience, heparin and insulin should be stored together in the refrigerator	False	1,842 (77.9)	522 (22.1)
5	Each drug better have multiple concentrations for nurse to choose	False	1,432 (60.6)	931 (39.4)
6	If a patient can tolerate, potassium can be administered orally instead of IV route	True	395 (16.7)	1,968 (83.8)
7	15% KCl is frequently used, so it should be easily and freely accessed by nurses	False	1,292 (54.7)	1,071 (45.3)
8	For pediatric dose, use teaspoon for dose expression	False	450 (19.0)	1,913 (81.0)
9	Fentanyl skin patch is a controlled medicine.	True	644 (27.3)	1,719 (72.7)
10	If a ward stores Atracurium for tracheal intubation, the drug should be stored with other drugs and easily accessed by nurses	False	1,753 (74.2)	610 (25.8)

### Overall Knowledge Related to High Alert Medications

Around 84% of the study participants achieved scores <70%, indicating majority of Pakistani nurses having poor knowledge of HAMs administration as well as regulation. Chi-square tests revealed that there was no significant difference of knowledge among different demographic variables (age, hospitals, departments, training, designations, qualification, and experience).

### Barriers Encountered During High Alert Medications Administration

Obstacles that nurses encounter during HAMs administration are shown in **Table 4**. Major barriers related to HAMs administration were “getting uncertain answers from colleagues” (72.9%), “unavailability of suitable person to consult” (61.1%), and “receiving verbal orders” (55.6%).

**Table 4:** Comparisons of high alert medications administration and high alert medications regulation scores among different demographic variables.

Variables	HAM administration score (mean rank)	p-value	HAM regulation score (mean rank)	p-value
**Age (years)**		0.797		0.867
≤25	1,184.45		1,194.81	
>25–30	1,185.95		1,169.03	
>30–35	1,191.47		1,180.65	
>35	1,148.13		1,199.26	
**Hospital type**		0.435		0.010
THQ hospital	1,204.50		1,199.15	
DHQ hospital	1,205.30		1,109.89	
Teaching hospital	1,168.33		1,207.21	
**Working department**		0.172		0.524
Cardiac	1,250.51		1,197.48	
Dialysis	1,143.54		1,241.20	
Emergency	1,145.29		1,174.59	
Gynea	1,196.02		1,159.08	
Labor room	1,209.24		1,034.19	
Medical	1,137.37		1,216.43	
Operation theatre	1,199.17		1,187.39	
Paeds	1,276.45		1,203.34	
Surgery	1,125.84		1,101.00	
**Training**		0.991		0.919
Basic life support	1,205.57		1,242.53	
Emergency room training	1,195.53		1,185.75	
Infection control	1,169.99		1,159.38	
Midwifery	1,209.46		1,177.07	
Operation theatre	1,197.45		1,147.25	
Personal protective equipment	1,158.82		1,169.46	
Nursery (Neonatal ICU)	1,156.17		1,146.07	
No training	1,177.75		1,191.74	
**Designation**		0.332		0.841
Staff nurse	1,178.88		1,182.64	
Head nurse	1,238.91		1,170.28	
**Qualification**		0.910		0.402
Bachelor	1,181.70		1,179.76	
Master	1,189.94		1,240.72	
**Experience (years)**		0.885		0.118
≤2	1,187.09		1,239.79	
>2–4	1,203.40		1,162.24	
>4–6	1,174.41		1,129.91	
>6–8	1,182.24		1,136.30	
>8–10	1,156.35		1,127.50	
>10	1,158.75		1,213.64	

DHQ, District Headquarter; HAM, High alert medication; ICU, Intensive care unit; THQ, Tertiary Headquarter.

## Discussion

This is the first ever study that provided insight not only about the knowledge of HAMs administration and regulation among Pakistani nurses but also the barriers encountered during administration of these medications. Our findings revealed that majority of nurses were found to have inadequate knowledge of HAMs which was comparable to the findings from Taiwan (Hsaio et al., 2010) and Palestine (Zyoud et al., 2019). Adrenaline should preferably administered *via* intramuscular route and IV route to be reserved only for extreme emergency in the presence of trained physician. Around one third of the nursing staff did not choose the right answer of adrenaline administration which was comparable to the findings of Hsaio et al. (2010) and Zyoud et al. (2019). Concentrated electrolyte solutions like KCl (15%), calcium chloride (CaCl_2_), and hypertonic saline should not be administer *via* IV push due to greater risk of complications. Incorrect IV administration of KCl (15%) causes adverse events like arrhythmias and cardiac arrest leading to patient’s death as documented in the previous studies (Reeve et al., 2005; Bonvin et al., 2009). Similarly, inappropriate administration of hypertonic saline cause phlebitis, extravasation injuries, and hypernatremia resulting in hypertensive emergencies especially in cardiac patients (Dillon et al., 2018). Administration of calcium salts *via* fast IV push is also associated with significant adverse events (Anger et al., 2014). The current study revealed that one third of the study population did not give correct response regarding 15% KCl and 10% CaCl_2_ administration, which was similar to the findings of a study conducted in Palestinian Nurses (Zyoud et al., 2019). Shockingly, around 39% of the study participants gave incorrect response about the dose calculation in children and cancerous patients. Likewise, Lan et al. also reported that knowledge of Taiwanese nurses about the dose calculation in aforementioned diseased population was poor (Lan et al., 2014).

While writing medication order for a particular patient, the clarity of written medication order is the key factor that can contribute to majority of mediations errors. Extreme caution should be taken while prescribing HAMs and defined units must be written in each medication instead of writing ampule or vial. Around 30% of the study participants were unaware of this regulation which was slightly better than the findings (correct response = 59.7%) of Hsaio and colleagues (2010). Zyoud et al. (2019) reported that around 20% of Palestinian nurses gave incorrect answer to question regarding the use of ampule or vial for dose expression instead of milligram or grams. Regarding the storage, HAMs must carry distinct labeling in order to differentiate them from rest of the medicines. Moreover, “heparin” and “insulin” should not be stored together in a single refrigerator due to chances of their mix up (Belknap, 2001). Around 22% of our nurses gave incorrect response to the aforementioned drugs which was similar to the findings of earlier studies (Hsaio et al., 2010; Lo et al., 2013; Zyoud et al., 2019). Similarly, atracurium can cause severe respiratory depression. Therefore, it must be separated from rest of the ward medicines in refrigerator, in separate box with clear labeling. Since spoons vary in sizes, they cannot be used to measure the accurate amount of medications. However, higher proportion (81%) of the study sample were unaware about the inappropriateness of spoon for pediatric dose measurement which was significantly higher than the findings of previous studies (Hsaio et al., 2010; Zyoud et al., 2019). Inadequacies in the knowledge of HAMs regulation and administration can be attributed to not obtaining extensive HAMs training. None of the study participants reported receiving extensive HAMs trainings in the present study which can be attributable to the severe shortage of clinical pharmacists in Pakistani health settings. Studies from Taiwan (mean 64.6 *vs* 54.6, p < 0.01) and Palestine (median 65 *vs* 50, p = 0.002) reported that HAMs trainings were associated with better knowledge (Hsaio et al., 2010; Zyoud et al., 2019). We strongly endorse the recommendation of Lo et al. (2013) regarding the inclusion of classes on the HAMs subject as a part of formal, in-school nursing education, as well as of hospital-based continuing education. Moreover, there is a dire need to bridge the gaps between physicians, clinical pharmacists, and nurses (**Table 5**) through effective communication. This will help reduce medication errors and improve patient care. Additionally, as there is a very high deficit of qualified and skilled healthcare workers, particularly nurses and midwives (FDGOP, 2019), Govt. should take measures necessary to improve the strength of nurses, doctors, and pharmacists in Pakistani hospitals.

**Table 5:** Barriers encountered during administration of high alert medication.

No.	Obstacles	N (%)
1	Insufficient knowledge	807 (34.1)
2	Have to accept oral order	1,314 (55.6)
3	Confused prescription	1,029 (43.5)
4	Inconsistent opinions between nurses	828 (35.0)
5	Inconsistent opinions between doctor and nurse	1,206 (51.0)
6	No reference for drug use	766 (32.4)
7	Receive uncertain answers from colleagues	1,724 (72.9)
8	Unclear dose calculation	910 (38.5)
9	No established standard operating procedures for high alert medications	867 (36.7)
10	No rigorous regulations for high alert medication	1,005 (42.5)
11	Mix high alert medications with other drugs	835 (35.3)
12	Easy access to high alert medications	898 (38.0)
13	No suitable person to consult	1,444 (61.1)

## Conclusions

The current study revealed the serious inadequacies in HAMs knowledge among Pakistani nurses which can cause medication errors leading to adverse patient outcomes. Major obstacles faced by nurses during HAMs administration were “getting uncertain answers from colleagues,” “unavailability of suitable person to consult,” and “receiving verbal orders.” Nursing students should receive comprehensive HAMs related education and training during graduation. Moreover, the training of the nursing staff should also be conducted on periodic basis. Each health facility in Pakistan must recruit sufficient number of medication experts (clinical pharmacists and pharmacovigilance officers) in order to ensure consultancy and availability of standard operation procedure for high alert medication safe storage, dispensing, and administration.

## Data Availability Statement

The datasets generated during and/or analyzed during the present study are available from the corresponding author (muhammad.salman@pharm.uol.edu.pk, msk5012@gmail.com) on reasonable request.

## Ethics Statement

This study was approved by the Research Ethics Committee of the Department of Pharmacy Practice, Faculty of Pharmacy, The University of Lahore. As the committee waived the need for written consent, a verbal informed consent was taken from every nurse before the enrollment.

## Author Contributions

All the authors contributed equally to this study.

## Conflict of Interest

The authors declare that the research was conducted in the absence of any commercial or financial relationships that could be construed as a potential conflict of interest.
