# Supplementary material for: Serious Inadequacies in High Alert Medication-Related Knowledge Among Pakistani Nurses: Findings of a Large, Multicenter, Cross-sectional Survey
Source: Front Pharmacol. 2020 Jul 14;11:1026. doi: 10.3389/fphar.2020.01026 (PMC7381221; doi:10.3389/fphar.2020.01026)
Supplement: Supplementary file 1 [file DataSheet_1.docx]

**APPENDIX: KNOWLEDGE ABOUT THE ADMINISTRATION AND REGULATION OF ‘HIGH ALERT MEDICATIONS’ AMONG PAKISTANI NURSES**

This questionnaire is administered to determine the knowledge about the administration and regulation of high-alert medication among registered Pakistani nurses. The study is purely for research purpose and all information provided will be treated with utmost confidentiality.

**Age:** _____________________ **years**

**Gender**: (A) Male (B) Female

**Name of Hospital: _____________________________________**_____________________

**Working Department:** _____________________

**Training (If any): _____________________________________**_____________________

**Experience:** _____________________

**Position/Designation: ______________________________**

**Degree:** ______________________

**Marital Status:** (A) Single (B**)** Married (C) Divorced/widow

**Residence:** (A) Rural (B) Urban

**Knowledge of High Alert Medications administration:**

| **S. No** | **Statement** | **True** | **False** | **Do not**  **Know** |
| --- | --- | --- | --- | --- |
| **1** | Fast intravenous push of 1:1000 epinephrine 1 ampule for patient who has mild allergic reaction |  |  |  |
| **2** | When an emergency happens, administer 10% calcium chloride (CaCl_2_) 10 ml as a fast intravenous push (1-2 minutes) |  |  |  |
| **3** | 10% calcium gluconate and 10% CaCl_2_ are the same drug and are interchangeable. |  |  |  |
| **4** | Dosage expression for insulin injection is ‘cc’ or ‘ml’ |  |  |  |
| **5** | Accurate chemotherapy dose calculation for adults is based on body weight whereas chemotherapy for children is based on body surface area |  |  |  |
| **6** | When an emergency such as ventricular fibrillation happens, push fast 15% potassium chloride (KCl) 10 ml intravenously |  |  |  |
| **7** | 15% KCl is better be added to Ringer’s solution for rapid infusion |  |  |  |
| **8** | Insulin syringe can be replaced by 1ml syringe |  |  |  |
| **9** | Fast IV infusion of 3% NaCl 500 ml for patient who has low sodium level |  |  |  |
| **10** | Port-A route can be used for blood withdrawal and drug injection generally |  |  |  |

**Knowledge of High Alert Medications Regulation**

| **S. No** | **Statement** | **True** | **False** | **Do not**  **Know** |
| --- | --- | --- | --- | --- |
| **1** | It is right to use ‘Amp’ or ‘Vial‘ for dose expression instead of ‘mg‘ or ‘gm’ |  |  |  |
| **2** | Distinctive labeling should be used on look-alike drugs |  |  |  |
| **3** | It is right to use ‘U’ instead of unit for dose expression” |  |  |  |
| **4** | For convenience, heparin and insulin should be stored together in the refrigerator |  |  |  |
| **5** | Each drug better have multiple concentrations for nurse to choose |  |  |  |
| **6** | If a patient can tolerate, potassium can be administered orally instead of IV route |  |  |  |
| **7** | 15% KCl is frequently used, so it should be easily and freely accessed by nurses |  |  |  |
| **8** | For pediatric dose, use teaspoon for dose expression |  |  |  |
| **9** | Fentanyl skin patch is a controlled medicine (regulated narcotic) |  |  |  |
| **10** | If a ward stores Atracurium for tracheal intubation, the drug should be stored with other drugs and easily accessed by nurses |  |  |  |

**What are the Barriers you encounter during administration of High Alert Medication?**

| **S. No** | **Statement** | **Yes** | **No** |
| --- | --- | --- | --- |
| **1** | I have insufficient knowledge. |  |  |
| **2** | I have to accept oral orders. |  |  |
| **3** | Confused prescription. |  |  |
| **4** | Inconsistent opinions between nurses. |  |  |
| **5** | Inconsistent opinions between doctor and nurse. |  |  |
| **6** | There is no reference for drug use. |  |  |
| **7** | I receive uncertain answers from colleagues. |  |  |
| **8** | Unclear dose calculation. |  |  |
| **9** | There is no established standard operating procedures for high alert medications. |  |  |
| **10** | There is no rigorous regulations for high alert medications. |  |  |
| **11** | I mix high alert medications with other drugs. |  |  |
| **12** | Easy access to high alert medications. |  |  |
| **13** | There is no suitable person to consult. |  |  |

Thank you for filling out this questionnaire.
